# An In Vitro Study of Protein S-Glutathionylation by Members of the CLIC Protein Family

**DOI:** 10.3390/biom15091213

**Published:** 2025-08-22

**Authors:** Wendy El Khoury, Khondker Rufaka Hossain, Amani Alghalayini, Hala M. Ali, Stella M. Valenzuela

**Affiliations:** School of Life Sciences, University of Technology Sydney, Sydney, NSW 2007, Australia; wendy.m.elkhoury@student.uts.edu.au (W.E.K.); khondker.hossain@uts.edu.au (K.R.H.); amani.alghalayini@uts.edu.au (A.A.);

**Keywords:** S-glutathionylation, deglutathionylation, glutathione, CLIC proteins, tryptophan quenching assay

## Abstract

Increasing evidence points to members of the chloride intracellular ion channel (CLIC) protein family performing a variety of functions within cells—classifying them as moonlighting proteins—and serving as natural cellular antioxidant protective agents. Apart from their role as membrane-inserting ion channels, members of the CLIC family also possess enzymatic oxidoreduction activity in their soluble form. The current study is the first to specifically examine the S-glutathionylation catalytic activity of several purified recombinant CLIC protein members (rCLIC1, rCLIC3, and rCLIC4) by directly measuring their ability to deglutathionylate and glutathionylate a synthetic model peptide via an in vitro tryptophan fluorescence quenching assay. Effects of pH and temperature on this activity were also assessed. Our findings provide insights into a likely previously uncharacterised mechanism by which CLIC proteins serve as cellular antioxidant protective enzymes via their S-glutathionylation capabilities.

## 1. Introduction

Protein S-glutathionylation is a critical post-translational modification involved in several processes, including modulation of signal transduction pathways and regulation of protein and enzymatic activity [1]. Importantly, it is also a means by which proteins can be protected from irreversible oxidative stress-induced damage caused by oxidation of protein thiols to sulfinic and sulfonic acids [1,2,3]. S-glutathionylation involves the formation of mixed disulfides with the addition of tripeptide glutathione (GSH) to protein cysteine residues, altering specific protein thiol groups post-translationally [1,4]. During cellular oxidative stress events, thiol groups in redox-sensitive proteins undergo reversible S-glutathionylation, which effectively caps these cysteine residues and protects them from permanent oxidative damage [5]. While this alteration can occur non-enzymatically, the involvement of particular enzyme thioltransferases from the glutathione S-transferase (GST) superfamily and the glutaredoxin (Grx) family are considered important mediators and thus contributors to cellular protective mechanisms [3].

The process of deglutathionylation, which removes the glutathione bound to proteins [3,6], is also catalysed by members of these protein thioltransferase families, including GSTomega 1-1 (GSTO1-1), Grx [7,8], thioredoxin (Trx), and sulfiredoxin (Srx) [1,9]. In general, proteins undergo enzymatic deglutathionylation once the cellular redox balance is restored, also emphasising the significance of glutathione in preserving cellular redox homeostasis [9,10,11,12,13]. Reversible S-glutathionylation has been associated with the regulation of numerous cellular processes, including apoptosis, cell cycle, inflammation, and actin contractile activity [9,10,14,15,16].

Since their discovery in the 1990s [17,18], members of the chloride intracellular ion channel (CLIC) protein family have been investigated in relation to a range of biologically significant pathways and disease processes. These include roles in cellular redox regulation and mitochondrial function, with CLIC4 and CLIC5 shown to localise to cardiac mitochondrial-associated membranes, where they modulate mitochondrial physiology [19,20]. Similarly, CLIC1 and CLIC4 were shown to regulate mitochondrial structure and biogenesis [21], and another study found CLIC4 was required to regulate redox homeostasis and mitochondrial function in breast cancer cells [22]. CLIC proteins have also been implicated in cellular ageing and immune responses, including cellular senescence in fibroblast [23] and endothelial [24] cells, and in antigen processing and presentation by dendritic cells [25]. Furthermore, they are also increasingly implicated in the progression of various pathological conditions, ranging from cardiovascular disorders [21] to pre-eclampsia [26], as well as in various cancers, ranging from hepatocellular carcinoma [27] and brain gliomas [28,29] to ovarian and breast cancers [30,31,32,33].

Most recently, studies by us and others provide support for the inclusion of members of the CLIC protein family as natural mediators of antioxidant processes in cells. These include a study on breast cancer cells, where CLIC4 was required to maintain mitochondrial function and resistance to exogenous oxidant assault [22]. Similarly, we demonstrated that the exogenous addition of purified recombinant protein—rCLIC1 or rCLIC4—to primary skin cells in culture protected against H_2_O_2_-induced oxidative damage, which was comparable to the protective actions of Grx and GSTO1-1 [34]. Furthermore, these studies coincide with structural [35,36,37,38] and computational studies [39,40] that place CLIC proteins as members within the larger GST protein superfamily, as well as studies showing the close structural resemblance of CLIC1 to the protein GSTO1-1 [35,39]. In addition, early structural studies of CLIC1 clearly identified the presence of an open slot or groove that includes its conserved GSH-binding site (including Cys^24^ in CLIC1 as the highly reactive thiol) [35]. This slot was proposed to serve as a substrate binding site for its target substrate [35], which could be an extended polypeptide chain.

As such, given the increasing evidence of the cellular protective capabilities of some CLIC proteins, and in order to explore a possible mechanism for these protective actions, the current study set out to investigate the glutathionylation and deglutathionylation activities of three purified recombinant proteins, CLIC1, CLIC3, and CLIC4, via an in vitro real-time assay and examine the effects of temperature and pH on these activities.

## 2. Materials and Methods

### 2.1. Reagents

The following reagents were purchased from Sigma Aldrich (Merck, Macquarie Park, NSW, Australia): glutathione reductase (GR) from yeast, reduced glutathione (GSH), nicotinamide adenine dinucleotide phosphate (NADPH), bovine plasma thrombin, kanamycin, isopropyl ß-D-1-thiogalactopyranoside (IPTG), tris(2-carboxyethyl) phosphine (TCEP), and indanyloxyacetic acid (IAA-94). The model peptides—SQLWCLSN and SQLWC(glutathione)LSN in both non-glutathionylated and glutathionylated forms—were custom-designed and purchased from Genescript Biotech (Nanjing, China). Recombinant GSTO1-1 protein (#GS75-100UG) was purchased from Sigma-Aldrich (Merck, Macquarie Park, NSW, Australia), and recombinant human glutaredoxin 1 protein (Grx 1) (#ab86987) was purchased from Abcam (Melbourne, VC, Australia). All other reagents used were of analytical grade.

### 2.2. Expression and Purification of Recombinant Proteins CLIC1, CLIC3, and CLIC4

Glycerol stocks of ClearColi^®^ BL21(DE3) Electrocompetent Cells (Astral Scientific, Taren Point, NSW, Australia) transformed with the His-tagged PET28a (+) expression vector (Novagen) containing the coding sequence for either human CLIC1 (NP_001279) or CLIC4 (NP_039234) were used to express recombinant CLIC1 and CLIC4 proteins. Glycerol stocks of *E. coli* BL21(DE3) cells transformed with the His-tagged PET28a (+) expression vector (Novagen, Sydney, NSW, Australia) containing the coding sequence for CLIC3 (NP_004660.2) were used to express the recombinant CLIC3 protein. The different CLIC proteins were expressed and purified as previously described [41]. Briefly, the recombinant CLIC proteins (rCLIC) were grown in 2xYT medium containing kanamycin at a concentration of 30 µg/mL (Sigma Aldrich) followed by induction with 1 mM IPTG (Sigma Aldrich) at OD_600nm_ and grown at 20 °C with overnight shaking at ~200 rpm. Cells were then harvested and resuspended in phosphate-buffered saline containing 0.5 mM TCEP prior to sonication. All soluble cell lysates were collected after an additional centrifugation at 10,000× *g* for 40 min at 4 °C and then subjected to affinity chromatography using Ni^2+^-NTA (Qiagen, Clayton, VC, Australia) column for His-tagged proteins. The His-tag was then removed by in-column thrombin enzymatic cleavage using an overnight incubation of bovine plasma thrombin (Sigma Aldrich) (30 NIH units per 1 L of bacterial culture) at 4 °C. The cleaved CLIC proteins were then collected in PBS buffer (10 mM phosphate buffer, 2.7 mM KCl, 140 mM NaCl, pH 7.4, and 0.5 mM TCEP) and further purified through size exclusion chromatography (SEC) (AKTA Pure/Amersham Pharmacia Biotech, NSW, Australia) using a HiPrep™ 16/60 Sephacryl^®^ S-100HR (Sigma Aldrich) column and equilibrated in column sizing buffer (100 mM KCl, 1 mM NaN_3_, 20 mM HEPES pH 7.5, and 0.5 mM TCEP). rCLIC1 and rCLIC4 proteins expressed in the ClearColi cells do not have endotoxins and were thus not subjected to endotoxin removal, whereas rCLIC3 proteins expressed in *E. coli* were subjected to endotoxin removal using the Pierce™ High Capacity Endotoxin Removal Spin Columns (ThermoFisher Scientific Cat # 88275, North Ryde, NSW, Australia) according to the manufacturer’s instructions. Furthermore, size exclusion chromatography helps in the removal of contaminants or impurities. The purity of rCLIC proteins was verified by SDS-Page gel electrophoresis and Western blotting using their respective anti-CLIC antibodies (Santa Cruz/Bio-Strategy PTY Limited, Melbourne, VC, Australia). Recombinant protein concentrations were measured spectrophotometrically and calculated using the extinction coefficient values of 0.647, 0.391, and 0.745 for CLIC1, CLIC3, and CLIC4, respectively, or by the Pierce^TM^ Bradford protein assay kit (ThermoFisher Scientific Cat # 23200) according to the manufacturer’s instructions. The purified samples were then aliquoted and stored at −80 °C for future experiments.

### 2.3. Assay for Determining Deglutathionylation/Glutathionylation

#### 2.3.1. Tryptophan Quenching Assay

The peptide substrates were used in both their non-glutathionylated and glutathionylated forms for fluorescence measurements, which were conducted using a Varian Cary Eclipse fluorospectrometer (Agilent Technologies, Mulgrave, VIC, Australia) with a 200 μL quartz cuvette.

The deglutathionylation assay was based on a previously described procedure that recorded the change in tryptophan fluorescence as glutathione was removed from a model peptide [7]. Fluorescence was recorded with an excitation wavelength of 280 nm, an emission wavelength of 356 nm, and slit widths set to 5 nm at room temperature over 500 s. For the deglutathionylation reaction, the reaction mix contained McIlvaine’s buffer (0.2 M disodium phosphate, 0.1 M citric acid, pH 7.0), 7 mM GSH, 50 μΜ NADPH, 0.25 units of glutathione reductase, 0.5 mM EDTA, 10 μM peptide, and 1 µM enzyme of interest. All measurements were recorded in triplicate.

Glutathionylation of the peptide was measured following the published method [7] with modification. The reaction mix was made in McIlvaine’s buffer (as above) with 5 mM GSSG, 0.5 mM EDTA, and 100 μM peptide, and between 0.1 and 0.2 µM of the enzyme of interest was added.

#### 2.3.2. IAA94 Blocker Studies

To further assess the activity of the recombinant proteins, CLIC1, CLIC3, and CLIC4 were pre-incubated with 10 μM IAA-94 (blocker drug) for 1 h at room temperature before being subjected to the respective assays. Protein concentrations were 1 μM for the deglutathionylation assay and 0.1 μM for the glutathionylation assay. The respective assays were then performed as described.

### 2.4. Analysis of Fluorescence Data

Raw fluorescence values were normalised by subtracting the background signal (buffer mixture control) from each test condition.

Data were expressed as the mean ± SEM and analysed using GraphPad Prism version 10.0.2 (GraphPad Software, La Jolla, CA, USA). Statistical significance was calculated using either one-way ANOVA or two-way ANOVA, followed by Tukey’s multiple comparisons test. All experiments were performed in triplicate except for the positive controls (commercially purchased recombinant proteins rGSTO1-1 and rGrx), which were run in duplicate. Statistical significance is indicated as follows: * *p* < 0.05, ns = not significant.

## 3. Results

### 3.1. In Vitro Deglutathionylation Assay Established for Positive Control Recombinant GSTO1-1

Previously, our group has demonstrated the glutathione-dependent oxidoreductase activity of several CLIC protein members [15] via a traditional indirect spectrophotometric coupled assay that measures the consumption of NADPH using the artificial non-specific substrate hydroxyethyl disulfide (HEDS). In the current study, we aimed to investigate if this oxidoreductase enzymatic activity is also applicable to protein substrates. We therefore used a modified version of the method developed by Peltoniemi et al. (2006) [8], “a new real-time method for measuring the deglutathionylation activity of glutaredoxins”. This assay utilises a glutathionylated peptide that contains a single glutathionylated cysteine residue adjacent to a tryptophan residue, which has intrinsic fluorescence via its aromatic ring structure. Reduction of glutathione (deglutathionylation of the peptide at the cysteine residue) results in an increase in total fluorescence with no shift in the emission maximum. We also included a non-glutathionylated version of the same peptide in order to study the glutathionylation capabilities of these three CLIC proteins. In a previous study by Menon and Board (2013) [7], the authors utilised this same tryptophan quenching assay to demonstrate GST-omega-1 (GSTO1-1) deglutathionylation activity. These previously published studies of Grx and GSTO1-1 employed 5 mM and 2 mM of GSH in the assay, respectively.

**Figure 1:**
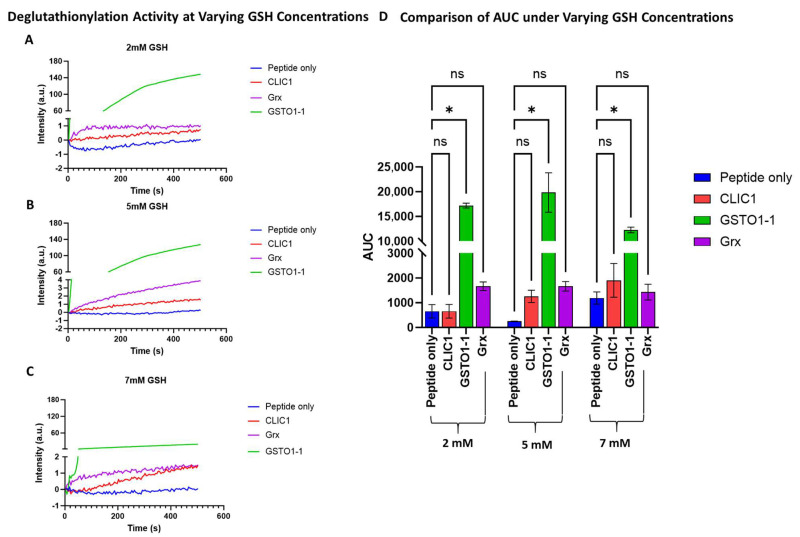
Comparison of deglutathionylation activity of CLIC1, Grx, and GSTO1-1 at different GSH concentrations. (**A**–**C**) A representative average intensity recording of the deglutathionylation activity measured by tryptophan quenching assay over a period of 500 s at 2 mM (**A**), 5 mM (**B**), and 7 mM (**C**) GSH concentrations for the three enzymes, compared to the peptide-only negative control sample. (**D**) Area under the curve (AUC) comparison of the deglutathionylation activities for CLIC1, GSTO1-1, and Grx following buffer subtraction and averaged across n = 4 repeats for the negative control and CLIC1 and n = 2 repeats for the positive controls GSTO1-1 and Grx. Statistical significance was assessed using two-way ANOVA followed by post hoc Tukey’s test. Error bars represent the standard error of the mean (SEM). Statistical significance is indicated as follows: * *p* < 0.05, ns = not significant.

In our current study, we included equimolar amounts [1 uM] of both rGrx and rGSTO1-1 as potential positive controls in the quenching assay, measuring their deglutathionylation activity and comparing [1 uM] rCLIC1 to these, with the peptide-only sample (no added enzyme) as the negative control. The initial use of 2 mM GSH resulted in little to no observable activity for both Grx and CLIC1, while GSTO1-1 displayed extremely high activity (Figure 1A), which was similar to the activity previously reported by Menon and Board (2013). Increasing GSH to 5 mM resulted in a slight increase in the deglutathionylation activity for CLIC1, with Grx also seen to be slightly active, while GSTO1-1 was found to show a relative decrease in overall activity. However, it retained significantly higher levels of activity compared to either CLIC1 or Grx (Figure 1B), with the latter two proteins showing more comparable levels of activity; however, this remained insignificant compared to the peptide-only control. By further increasing GSH to 7 mM, CLIC1 activity remained the same (1.5 a.u), while both Grx and GSTO1-1 showed a relative decrease in their respective activities (Figure 1C). Higher concentrations of GSH resulted in reduced activity of CLIC proteins. Therefore, the initial assay conditions used were found to be optimal for rGSTO1-1; however, no significant activity was detected for either rGrx or rCLIC1.

In all subsequent experiments, 7 mM GSH was used as the standard concentration for the tryptophan quenching assays.

### 3.2. Recombinant CLIC1, CLIC3, and CLIC4 Show No Discernible In Vitro Deglutathionylation Activity at pH 7

**Figure 2:**
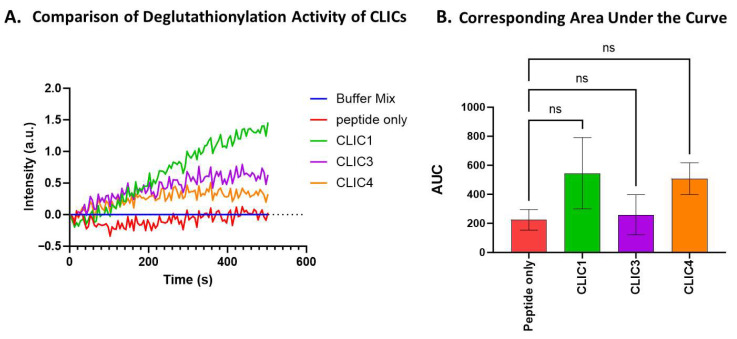
Comparison of the deglutathionylation activity and the corresponding AUC of CLIC1, CLIC3, and CLIC4 shows no significant activity. (**A**) Representative trace of the deglutathionylation activity measured by fluorescence intensity over time for CLIC1, CLIC3, and CLIC4. (**B**) Area under the curve (AUC) comparison of deglutathionylation activities for CLIC1, CLIC3, and CLIC4. Repeats n = 6. Statistical significance was assessed using one-way ANOVA followed by post hoc Tukey’s test. Error bars represent the standard error of the mean (SEM). Statistical significance is indicated as follows: ns = not significant.

The proteins CLIC3 and CLIC4 were also assayed under the same deglutathionylation assay conditions as above using 7 mM GSH. While the three CLIC proteins, CLIC1, CLIC3, and CLIC4, all showed comparable levels of activity (Figure 2A), resulting in AUC values of 545, 258, and 507, respectively, this was not deemed to be a significant difference from the peptide-only control (Figure 2B) with an AUC value of 224 ± 160 (a.u).

### 3.3. Recombinant CLIC4 Deglutathionylation Activity Found to Be pH-Dependent

**Figure 3:**
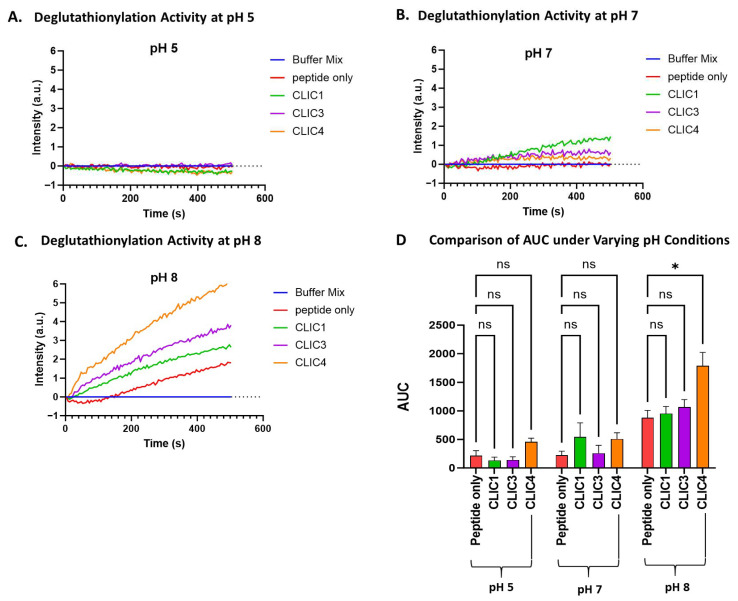
pH effect on CLIC proteins’ deglutathionylation activity. Tryptophan quenching assay using recombinant CLIC proteins at pH 5 (**A**), pH 7 (**B**), and pH 8 (**C**). (**D**) AUC comparison of recombinant CLIC proteins with the peptide-only negative control at pH 5, 7, and 8. Statistical significance was assessed using two-way ANOVA followed by post hoc Tukey’s test. Error bars represent the standard error of the mean (SEM). Statistical significance is indicated as follows: * *p* < 0.05, ns = not significant. n = 5.

pH is a critical parameter to which many cellular processes, including the actions of proteins and enzymes, are extremely sensitive. Previously, studies of human CLIC1 ion channel activity have shown its activity increases under lower pH conditions [42,43], and a similar effect was found for the bacterial CLIC homolog, stringent starvation protein A (SspA) [44]. Not surprisingly, the oxidoreductase activity of CLIC proteins in the HEDS assay was also found to be pH-sensitive [41]. Therefore, the impact of pH on CLIC proteins’ enzymatic activity in the tryptophan quenching assay was assessed by running the assay with McIlvaine’s buffer adjusted to pH 5, 7, or 8. As seen in Figure 3, all three proteins demonstrated some apparent pH sensitivity. The enzymatic functioning of CLIC1, CLIC3, and CLIC4 appears to occur optimally under more alkaline conditions, with all three proteins showing their highest deglutathionylation activity at pH 8, with an increasing trend in their activity observed; however, only CLIC4’s activity was deemed significantly different compared to the peptide-only control at pH 8, resulting in an AUC value of 1800 ± 521 (a.u), which was approximately a 2-fold increase in activity compared to the peptide-only control (AUC value 880 ± 316).

### 3.4. Deglutathionylation Activity of Recombinant CLIC Proteins Attenuated by Synthetic CLIC Protein Antagonist, IAA-94

**Figure 4:**
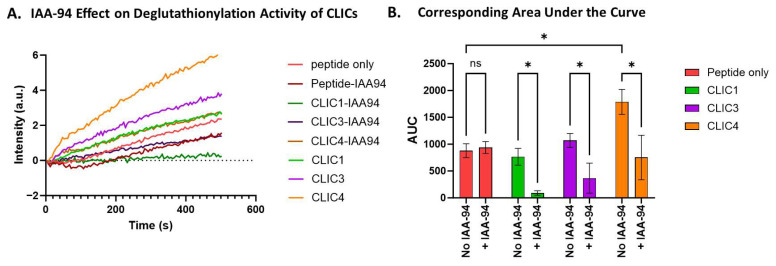
IAA94 effect on CLIC proteins’ deglutathionylation activity. (**A**) Tryptophan quenching assay using recombinant CLIC proteins incubated with IAA94 at pH 8. (**B**) AUC comparison of recombinant CLIC protein activity in the absence and presence of the blocker IAA-94 at pH 8 (repeats n = 3). Statistical significance was assessed using two-way ANOVA followed by post hoc Tukey’s test. Error bars represent the standard error of the mean (SEM). Statistical significance is indicated as follows: * *p* < 0.05, ns = not significant.

To assess whether the chloride channel inhibitor IAA-94 affects the deglutathionylation activity of rCLIC proteins, we incubated CLIC1, CLIC3, and CLIC4 with 10 μM IAA-94 for 1 h on ice prior to running the assay at pH 8, as this is the condition that showed significant deglutathionylation activity for only CLIC4 (Figure 3). As seen in Figure 4, the presence of IAA-94 significantly attenuated the deglutathionylation activity of CLIC4. In comparison to the peptide-only control, CLIC4 demonstrated a 2-fold increase in AUC value, whereas CLIC4 activity was attenuated 0.5-fold in the presence of the blocker, thus resulting in a significant decrease in its activity. Although CLIC1 and CLIC3 did not show a significant level of deglutathionylation activity under any pH conditions, incubation with IAA-94 did show a reduction in their presumed activity, while the addition of the drug IAA-94 had no effect on the peptide-only control (no enzyme added).

### 3.5. Equivalent In Vitro Glutathionylation by Recombinant CLIC1, Grx, and GSTO1-1

Whilst CLIC1 was found to not have significant deglutathionylation activity under the conditions tested, its glutathionylation activity was then tested and compared to Grx and GSTO1-1 using the non-glutathionylated form of the model SQLWCLSN peptide in the presence of oxidised glutathione (GSSG). Equimolar amounts [0.1 uM] of each recombinant protein were used following the method outlined in [7]. The reaction without the addition of enzyme was used as the negative control to determine the baseline rate of glutathionylation.

**Figure 5:**
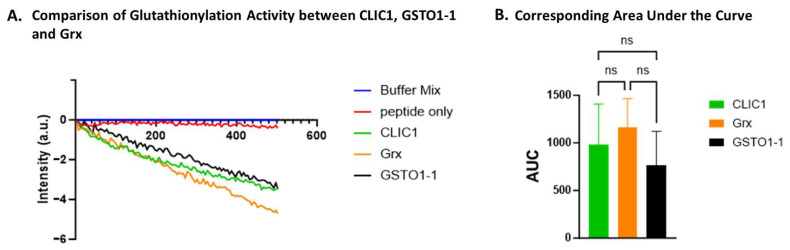
Glutathionylation activity of CLIC1. (**A**) A representative trace demonstrating glutathionylation activity measured by fluorescence intensity over time for CLIC1, Grx, and GSTO1-1. (**B**) Area under the curve (AUC) comparison of glutathionylation activities for CLIC1, Grx, and GSTO1-1. Statistical significance was assessed using one-way ANOVA followed by post hoc Tukey’s test. Error bars represent standard deviation (SEM). Statistical significance is indicated as follows: ns = not significant. n = 3.

A significant decrease in fluorescence intensity compared to the peptide-only control was observed for all three samples (Figure 5A) where enzyme had been added, including rCLIC1 and the two positive controls, Grx and GSTO1-1, indicating successful glutathionylation of the peptide. Although the activity of all three proteins against the peptide control was found to be significantly different, there was no statistically significant difference in activity between the three proteins CLIC1, Grx, and GSTO1-1 (Figure 5B).

### 3.6. In Vitro Glutathionylation by Recombinant CLIC1, CLIC3, and CLIC4

**Figure 6:**
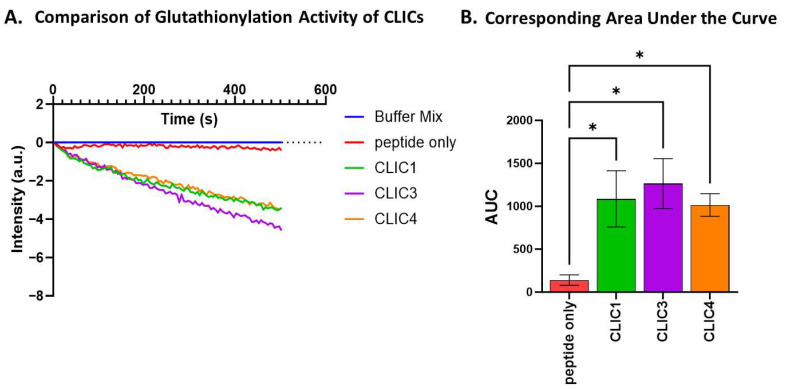
Glutathionylation activity of CLIC1, CLIC3, and CLIC4. (**A**) Glutathionylation activity measured by fluorescence intensity over time for CLIC1, CLIC3, and CLIC4. (**B**) Area under the curve (AUC) comparison of glutathionylation activities for CLIC1, CLIC3, and CLIC4. Statistical significance was assessed using one-way ANOVA followed by post hoc Tukey’s test. Error bars represent standard error of the mean (SEM). Statistical significance is indicated as follows: ns = not significant, * *p* < 0.05. n = 4.

rCLIC1’s glutathionylation activity was then compared to rCLIC3 and rCLIC4 using equimolar amounts of each protein [0.1 uM]. All three rCLIC proteins showed comparable glutathionylation activity via an observed reduction in fluorescence intensity (Figure 6A). AUC analysis showed a significant 7-fold increase in the glutathionylation activity for all three tested CLIC proteins, resulting in an AUC value of between 1100 and 1300 (a.u) compared to the negative control (AUC value of 150 ± 100 a.u), but there was no significant difference in their activity when compared to each other (Figure 6B).

### 3.7. Comparison of the Three Recombinant CLICs’ Glutathionylation Activity Shows Some Temperature Sensitivity Differences

**Figure 7:**
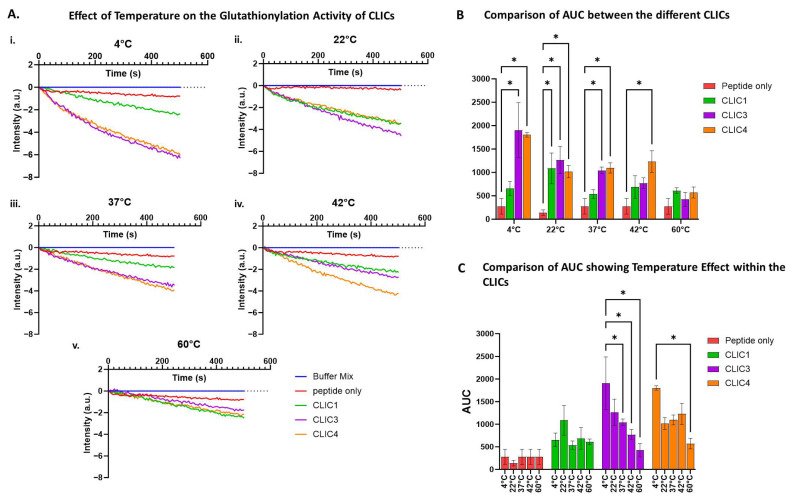
Effects of pre-heating or cooling of CLIC proteins on their glutathionylation activity. (**A**) Glutathionylation activity measured by fluorescence intensity over time after pre-heating/cooling CLIC proteins at +4 °C (**i**), 22 °C (**ii**), 37 °C (**iii**), 42 °C (**iv**), or 60 °C (**v**) across different temperature ranges for 10 min before subjecting the proteins to the tryptophan quenching assay. (**B**) Area under the curve (AUC) comparison of glutathionylation activities for CLIC1, CLIC3, and CLIC4 with the peptide-only control following pre-heating at different temperatures before the tryptophan quenching assay. (**C**) Area under the curve (AUC) comparison of glutathionylation activities of CLIC proteins over different temperature ranges. Statistical significance was assessed using two-way ANOVA followed by post hoc Tukey’s test. Error bars represent the standard error of the mean (SEM). Statistical significance is indicated as follows: * *p* < 0.05, n = 3.

We used the tryptophan quenching assay to investigate how exposure to different temperatures would impact the three purified rCLIC proteins’ glutathionylation activity. The three rCLIC proteins were pre-heated or cooled for 10 min at various temperatures ranging between +4 °C and 60 °C immediately prior to their addition into the assay, which was run at room temperature. As seen in Figure 7, rCLIC1 appears to be the least temperature-sensitive of the three proteins, with rCLIC3 seen to be the most sensitive, especially at higher temperatures. rCLIC3 and rCLIC4 showed greater activity when kept at +4 °C prior to use. Pre-heating to +60 °C resulted in reduced activity for both rCLIC3 and rCLIC4.

According to previously published studies, the deglutathionylation/glutathionylation reaction for GSTO1-1, as measured by the tryptophan quenching assay, is optimal at room temperature and pH 7 [7,8]. Our data demonstrates that CLIC protein incubation temperature affects their S-glutathionylation activity, with room temperature (approximately 22 °C) for CLIC1 or storage on ice (+4 °C) for CLIC3 and CLIC4 being the most favourable conditions for maintaining most of the CLICs’ enzymatic activity. These proteins may maintain a conformational structure more conducive to catalytic activity at 4 °C or 22 °C, as most enzymes often exhibit folding dynamics that are temperature-dependent. Higher temperatures, such as 42 °C and 60 °C, reduce the proteins’ activity by likely destabilising their structure and/or leading to their denaturation.

### 3.8. pH Dependence of Glutathionylation by Recombinant CLIC3 and CLIC4

**Figure 8:**
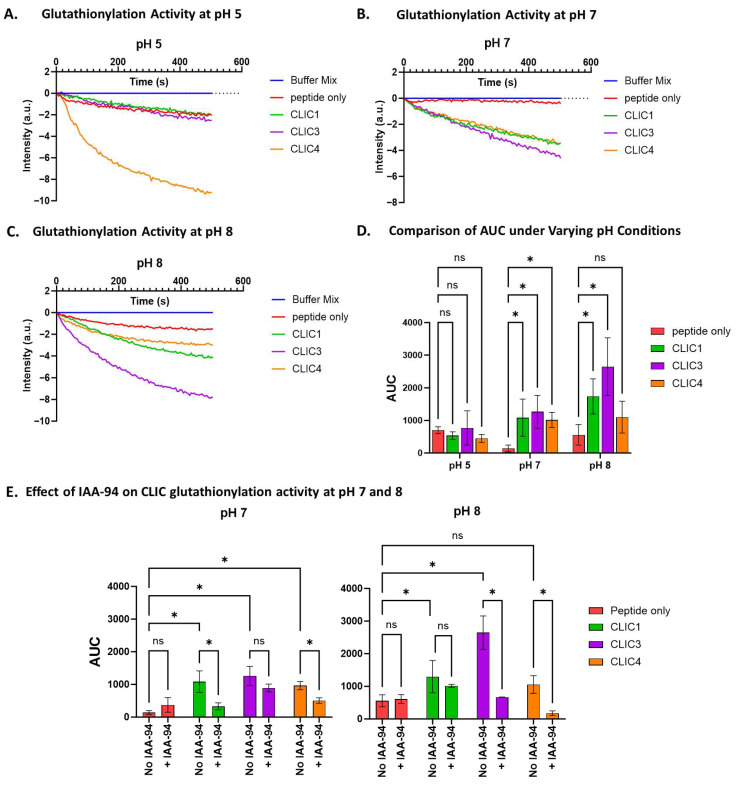
pH effect on CLIC proteins’ glutathionylation activity. Tryptophan quenching assay using recombinant CLIC proteins and peptide-only control at pH 5 (**A**), pH 7 (**B**), and pH 8 (**C**). (**D**) AUC comparison of recombinant CLIC proteins at pH 5, 7, and 8. (**E)** AUC comparison of recombinant CLIC proteins and CLIC-IAA94 at pH 7 and 8. Statistical significance was assessed using two-way ANOVA followed by post hoc Tukey’s test. Error bars represent the standard error of the mean (SEM). Statistical significance is indicated as follows: * *p* < 0.05, ns = not significant. n = 4.

As noted in the earlier experiments, different pH levels affect the deglutathionylation activity of rCLIC proteins. Here, we investigated the effect of different pH levels—pH 5, 7, and 8—on the glutathionylation activity of three rCLIC proteins. As seen in Figure 8D, the glutathionylation activity of all three proteins was significantly affected by the pH variations when averaged over several repeat experiments. None of the three proteins showed significant activity at pH 5 compared to the peptide-only control. At pH 7, all three were active, resulting in approximately a 7-fold increase in activity, while CLIC1 and CLIC3 showed the highest activity at pH 8, resulting in an increase in activity by 20% for CLIC1 and almost 70% for CLIC3 in comparison to pH 7. Interestingly, CLIC4 was found to not have significant activity at pH 8, which contrasts with CLIC4 showing deglutathionylation activity at pH 8.

To further verify the glutathionylation activity of rCLIC proteins at varying pH, each of the recombinant proteins was pre-incubated with 10 μM IAA-94 for 1 h, and their glutathionylation activity was assayed across different pH levels. The drug showed significant attenuation of rCLIC activity for all three proteins, but not surprisingly, this varied depending on the specific CLIC protein and the pH condition (Figure 8E). Pre-incubation with the blocker resulted in CLIC1 showing significant inhibition at pH 7, resulting in a 0.7-fold decrease in its glutathionylation activity, from an AUC value of 1086 ± 500 to 329 ± 182 a.u, while CLIC4 showed a 0.4-fold decrease in activity. CLIC3 activity, on the other hand, was notably reduced but not significantly inhibited at pH 7. At pH 8, although CLIC1 showed significant activity, this was not significantly inhibited by the blocker, whereas CLIC3 activity was significantly reduced by 0.2-fold. Although CLIC4 showed no significant activity at pH 8 compared to the peptide-only control, it was found to be inhibited by the blocker. This strongly suggests that the activity of both rCLIC proteins and their interactions with the blocker are highly pH-dependent.

## 4. Discussion

S-glutathionylation is an essential post-translational modification involved in regulating cellular signalling and redox control [2,5]. Our goal in this study was to clarify the deglutathionylation/glutathionylation capabilities of three CLIC protein family members, CLIC1, CLIC3, and CLIC4, via an in vitro tryptophan quenching assay using a model peptide substrate. The combined results presented in this study demonstrate that all three proteins have S-glutathionylation capabilities as part of their known general oxidoreductase enzymatic activity [45]. Their capacity to carry out these enzymatic reactions on protein substrates, in turn, supports a role for CLIC proteins in cellular redox processes and preservation of protein thiol redox states, as well as their consideration as members of the natural cellular antioxidant protective enzyme arsenal.

IAA94 is known to block both ion channel [41] and non-channel functions of CLIC proteins [45]. In our assay, IAA94 inhibited the activity of all three CLIC proteins in a pH-dependent manner. These findings suggest that the thiol reactivity or structural conformation of CLIC proteins plays a critical role in their enzymatic function.

pH clearly impacted the S-glutathionylation activity of the three CLIC proteins. CLIC4 demonstrated a deglutathionylation activity under more alkaline conditions (pH 8), while CLIC1 and CLIC3 were not found to have any significant deglutathionylation activity under the conditions studied. On the other hand, all three CLIC proteins demonstrated significant glutathionylation activity at pH 7 but no activity at pH 5. While CLIC1 and CLIC3 showed their highest significant activity at pH 8, CLIC4 showed no glutathionylation activity at pH 8. Such pH dependencies—which were also previously reported for CLIC proteins when tested in the HEDS assay [45]—may serve as a means of regulating individual CLIC protein enzymatic activity via local intra/extracellular milieu conditions.

The limitations of this study, however, need to be considered. Even though in vitro assay systems provide useful controlled means to study enzymatic activity, one does need to be mindful of the limitations of using model synthetic substrates and artificial enzyme reaction conditions. These likely do not reflect the true nature and complexity of the binding interactions of an enzyme with its native protein substrate, nor do they effectively mimic the more complex biological milieu within which such reactions would be occurring. Furthermore, having now confirmed that all three of these CLIC proteins have S-glutathionylation capabilities, the task remains to identify the specific protein substrate(s) targeted by each CLIC protein. Future studies to be pursued include the use of CLIC knockdown cell lines, combined with Western blot analysis using an antibody that recognises GSH bound to proteins, to identify potential protein targets for CLIC proteins. This approach was used to successfully demonstrate beta-actin as a specific target for GSTO1-1’s S-glutathionylation activity [7].

A study of the secretome from cancer-associated fibroblasts found that CLIC3 was abundantly expressed. CLIC3 was shown to post-translationally modify the protein transglutaminase-2 (TGM2) by reducing it in the presence of GSH, thus regulating TGM2 activity [30]. This in turn served to drive angiogenesis and cancer progression by promoting TGM2-dependent invasion [30]. This clearly demonstrates CLIC3 protein involvement in the glutathionylation cycle as a regulator of cell signalling processes. We now also extend these activities to include antioxidant protective functions in cells, based on our current in vitro biochemical study demonstrating their protein S-glutathionylation capabilities and recent cell studies by us [34] and others [22] demonstrating their cellular protective capabilities.

## 5. Conclusions

This study demonstrates that CLIC1, CLIC3, and CLIC4 proteins have S-glutathionylation capabilities as part of their known general oxidoreductase enzymatic activity, which was also found to be pH-dependent. Their capacity to carry out these enzymatic reactions on protein substrates provides a mechanism that supports a role for CLIC proteins in cellular redox processes and preservation of protein thiol redox states, as well as their consideration as members of the natural cellular antioxidant protective enzyme arsenal. It also further supports their classification as moonlighting proteins capable of acting as both soluble enzymes and membrane ion channel proteins.

## Data Availability

The original contributions presented in this study are included in the article material. Further enquiries can be directed to the corresponding author.
